# The novel long intergenic noncoding RNA *UCC* promotes colorectal cancer progression by sponging miR-143

**DOI:** 10.1038/cddis.2017.191

**Published:** 2017-05-11

**Authors:** Feng-Ting Huang, Wen-Ying Chen, Zhi-Qiang Gu, Yan-Yan Zhuang, Chu-Qiang Li, Ling-Yun Wang, Juan-Fei Peng, Zhe Zhu, Xin Luo, Yuan-Hua Li, He-Rui Yao, Shi-Neng Zhang

**Affiliations:** 1 Department of Gastroenterology and Guangdong Provincial Key Laboratory of Malignant Tumor Epigenetics and Gene Regulation, Sun Yat-sen Memorial Hospital, Sun Yat-sen University, Guangzhou, Guangdong Province, China; 2 Department of Gastroenterology, the Fifth Affiliated Hospital, Sun Yat-sen University, Zhuhai, Guangdong Province, China; 3 Department of Stem Cell Biology and Regenerative Medicine, Cleveland Clinic, Lerner Research Institute, Cleveland, OH, USA; 4 Breast Tumor Center and Guangdong Provincial Key Laboratory of Malignant Tumor Epigenetics and Gene Regulation, Medical Research Center, Sun Yat-sen Memorial Hospital, Sun Yat-sen University, Guangzhou, Guangdong Province, China

## Abstract

The human genome contains thousands of long intergenic noncoding RNAs (lincRNAs). However, the functional roles of these transcripts and the mechanisms responsible for their deregulation in colorectal cancer (CRC) remain elusive. A novel lincRNA termed upregulated in CRC (*UCC*) was found to be highly expressed in human CRC tissues and cell lines. *UCC* levels correlated with lymph node metastasis, Dukes’ stage, and patient outcomes. In SW480 and SW620 cells, knockdown of *UCC* inhibited proliferation, invasion, and cell cycle progression and induced apoptosis *in vitro.* Xenograft tumors grown from *UCC*-silenced SW620 cells had smaller mean volumes and formed more slowly than xenograft tumors grown from control cells. Inversely, overexpression of *UCC* in HCT116 promoted cell growth and invasion *in vitro*. Bioinformatics analysis, dual-luciferase reporter assays, and RNA immunoprecipitation assays showed that miR-143 can interact with *UCC*, and we found that *UCC* expression inversely correlates with miR-143 expression in CRC specimens. Moreover, mechanistic investigations showed that *UCC* may act as an endogenous sponge by competing for miR-143, thereby regulating the targets of this miRNA. Our results suggest that *UCC* and miR-143 may be promising molecular targets for CRC therapy.

Worldwide, colorectal cancer (CRC) is the third most commonly diagnosed cancer in males and the second most commonly diagnosed cancer in females, and an estimated 1.4 million CRC cases and 693 900 CRC-related deaths occurred in 2012.^1^ Understanding the molecular mechanisms that govern tumor growth and metastasis is imperative for establishing early detection strategies as well as individualized treatment. Molecular analysis has enabled the development of diagnostic and therapeutic tools facilitating precision medicine that has previously been unavailable.^2, 3^ Although previous studies have documented that alterations in many oncogenes and tumor-suppressor genes are associated with CRC, the molecular and genetic bases of colorectal carcinogenesis remain largely unknown.^4^

The human transcriptome contains not only many protein-coding messenger RNAs (mRNAs) but also a large set of non-protein-coding transcripts that have structural, regulatory, or unknown functions. Recent studies have revealed that the human genome encodes many noncoding RNAs ranging from small regulatory RNAs such as microRNAs and Piwi-associated RNAs to long noncoding RNAs (lncRNAs, longer than 200 nucleotides). The exact number of lncRNAs encoded by the human genome is a matter of debate, but most estimates place the number in the tens of thousands.^5, 6^ Long intergenic noncoding RNAs (lincRNAs), a type of lncRNAs, are transcript units that discretely intervening between known protein-coding loci. Although the functions of a few lincRNAs, such as XIST and HOTAIR, have been characterized in some important cellular processes, such as X chromosome inactivation, genomic imprinting, pluripotency maintenance, and transcriptional regulation,^7, 8^ the functions of most annotated lincRNAs remain unexplored. However, several studies have implicated lincRNAs in a variety of disease states, including cancers.^9, 10, 11^ Recent studies have demonstrated that several lincRNAs are involved in the tumorigenesis and development of CRC.^12, 13^ However, an enormous number of lincRNAs remain to be elucidated and characterized.

In this study, differences in the lincRNA expression profiles between CRC and tumor-adjacent nontumor tissues were assessed via lincRNA expression microarray analysis, and we observed 124 dysregulated lincRNAs and 1583 dysregulated mRNAs in CRC samples. Among the upregulated lncRNAs, we characterized the pathologic relevance of lincRNA ENST00000602992 (which we termed upregulated in colorectal cancer, *UCC*) in CRC growth and progression. First, we measured the levels of *UCC* transcripts in CRC tissues and cell lines and confirmed the upregulation of *UCC* in CRC. The expression of *UCC* closely correlated with lymph node metastasis, Dukes’ stage and overall survival. Furthermore, we identified a role of *UCC* in CRC cell growth and metastasis based on *in vitro* and *in vivo* functional experiments. Finally, mechanistic investigations revealed that *UCC* can promote CRC progression by acting as a sponge for miR-143, which is known to have a key role in diverse physiological and pathological processes.^14, 15, 16^ Taken together, these results suggest that *UCC* and miR-143 may be promising molecular targets for CRC therapy.

## Results

### The novel lincRNA *UCC* is upregulated in CRC

To identify lincRNAs that are dysregulated in CRC, we employed a lincRNA microarray analysis covering 27 958 protein-coding transcripts and 7419 annotated and/or known lincRNAs (Agilent). Filtered by *P*-value and fold change (*P*<0.01 and fold change >2 or fold change <0.5 for lincRNAs/ mRNAs), a total of 124 lincRNAs and 1583 mRNAs were differentially expressed between four paired CRC and non-tumor tissues. Hierarchical clustering showed systematic variations in transcript expression levels between the paired tumor and non-tumor tissues (Supplementary Figures S1A and B). Gene Ontology (GO) and pathway analyses indicated that most differentially expressed genes were involved in cell proliferation as well as cell death control (Supplementary Figure S2). The top 30 differentially expressed lincRNAs are provided in Supplementary Table S1. The microarray data mentioned in this article are available in the *National Center for Biotechnology Information* Gene Expression Omnibus under accession number GSE75970.

We primarily focused on upregulated lincRNAs because this set of lincRNAs can be used more readily than downregulated lincRNAs as early diagnostic markers or therapeutic targets. We chose four overexpressed lincRNAs with fold changes in expression >2 based on microarray analysis and validated the expression results in an additional eight pairs of CRC and non-tumor tissues. *UCC* was the most highly upregulated lincRNA in CRC tissues compared to non-tumor tissues (Supplementary Figures S1C, S3 [Supplementary-material sup1]and Supplementary Table S1). Information from the UCSC Genome Browser shows that *UCC* is a 747-bp transcript with one exon and localizes in human chromosome 7p15.2 (Supplementary Figure S1D).

### *UCC* expression correlates with CRC progression

**Figure 1:**
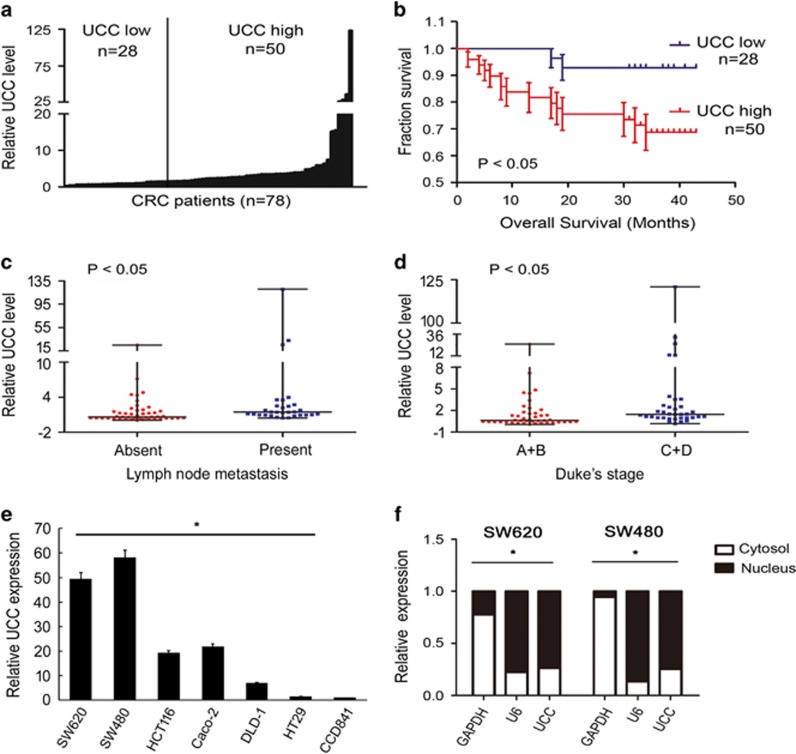
*UCC* expression correlates with CRC progression. (**a**) *UCC* expression in CRC tissues from 78 cases based on qRT-PCR analysis. The high value of *UCC* was defined as fold change >2 (*n*=50), the rest including downregulation or no evident difference in expression in CRC tissues compared with *UCC* expression in the paired non-tumor tissue, was defined as low values (*n*=28). (**b**) Kaplan–Meier curves of the survival of 78 patients were evaluated using the log-rank test. (**c**) *UCC* expression in the lymph node metastasis-negative group (*n*=46) and the lymph node metastasis-positive group (*n*=32). (**d**) *UCC* expression in CRC tissues from different Dukes’ stages: stage A+B (*n*=43) and stage C+D (*n*=35). Mann–Whitney test was used to analyzed the differences between groups in **c** and **d**, data were presented as the median with range. (**e**) Abundance of *UCC* in CRC cell lines relative to that in the colonic epithelial cell line CCD841. The expression of *UCC* was normalized to that in CCD841. The statistical differences between groups were analyzed using independent samples *t*-test. Error bars represent the mean±S.D. of triplicate experiments. *P<0.05. (**f**) Cellular localization of *UCC* in CRC cells. *GAPDH* and *U6* serve as a cytoplasmic and nuclear localization marker, respectively

Then, we examined levels of *UCC* in CRC tissues obtained from 78 independent patients at Sun Yat-sen Memorial Hospital of Sun Yat-sen University (Guangzhou, China) using quantitative real-time PCR (qRT-PCR). *UCC* expression in CRC tissues was increased in 50 cases (64%), whereas 28 cases (36%) showed downregulation or no evident difference in expression in CRC tissues compared with *UCC* expression in the paired non-tumor tissue (Figure 1a). Kaplan–Meier analysis suggested a positive correlation between tumoral *UCC* expression and a significantly reduced overall survival time among CRC patients with upregulated *UCC* expression compared to CRC patients without upregulated *UCC* expression (*P*<0.05, Figure 1b). High levels of *UCC* were also found in patients with lymph node metastasis and advanced Dukes’ stage (Figures 1c, d and Table 1). Consistently, *UCC* was upregulated in CRC cell lines (Figure 1e) and preferentially localized to the nucleus (Figure 1f). Taken together, these data show that *UCC* is indeed highly expressed in CRC in association with cancer progression.

### Knockdown of *UCC* inhibits CRC cell growth and invasion

**Figure 2:**
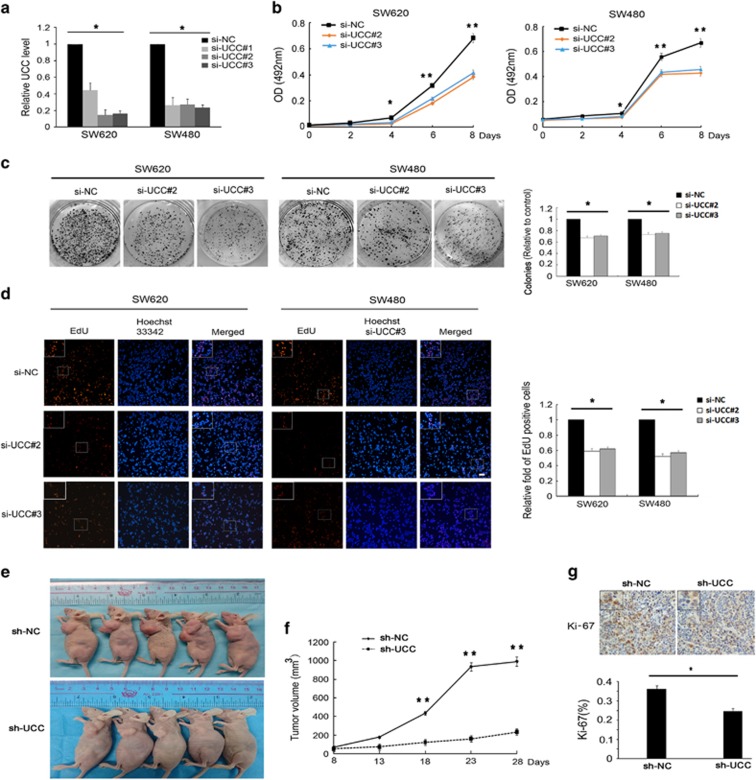
*UCC* knockdown inhibits CRC cell growth *in vitro* and *in vivo*. (**a**) *UCC* expression levels were suppressed by specific siRNAs in CRC cells. (**b**) Growth curves of SW620 and SW480 cells after transfection with si-*UCC* or si-NC were determined via MTS assays. (**c**) The anchorage-independent growth of SW620 and SW480 cells was assessed via colony formation assays. (**d**) Cell proliferation was evaluated using EdU incorporation assays. Proliferating cells were labeled with EdU. Scale bar: 200 *μ*m. (**e**) Effects of *UCC* knockdown on tumor growth after 4 weeks *in vivo* (*n*=5 per group). Upper: negative control cells. Lower: representative images of tumors formed in nude mice subcutaneously injected with *UCC*-silenced SW620 cells. (**f**) Growth curves of xenograft tumors after subcutaneous injection of mice with *UCC*-silenced SW620 or negative control cells. The tumor volumes were measured every 5 days after inoculation. (*n*=5). (**g**) Immunohistochemical staining showed that *UCC* knockdown decreased the Ki-67 proliferation index. The data were represented as the mean±S.D. of three independent experiments *in vitro* or five independent experiments *in vivo*. *P<0.05, **P<0.01 by Student’s *t*-test

To evaluate the possible role of *UCC* in CRC, we transfected SW620 and SW480 cells with three different siRNAs against *UCC* (designated si-*UCC*#1-3), all of which efficiently knocked down the endogenous *UCC* level (Figure 2a). To avoid off-target effects, we chose si-*UCC*#2 and si-*UCC*#3 for subsequent experiments. The results of the CellTiter 96 AQueous One Solution Cell Proliferation Assay (MTS) indicated that silencing *UCC* reduced the viability of SW620 and SW480 cells (Figure 2b). Moreover, colony formation assays and EdU incorporation assays showed that *UCC* knockdown significantly inhibited the proliferative capacity of SW620 and SW480 cells (Figures 2c and d). These suppressive effects were confirmed by *in vivo* tumor growth assays. Xenograft tumors grown from *UCC*-silenced SW620 cells had smaller mean volumes and formed more slowly than xenograft tumors grown from control cells (Figures 2e and f). In addition, positive staining for the proliferation marker Ki-67 was significantly decreased in *UCC*-silenced cells compared to control cells (Figure 2g). Collectively, these data implied that suppression of *UCC* expression contributed to CRC cell growth inhibition.

**Figure 3:**
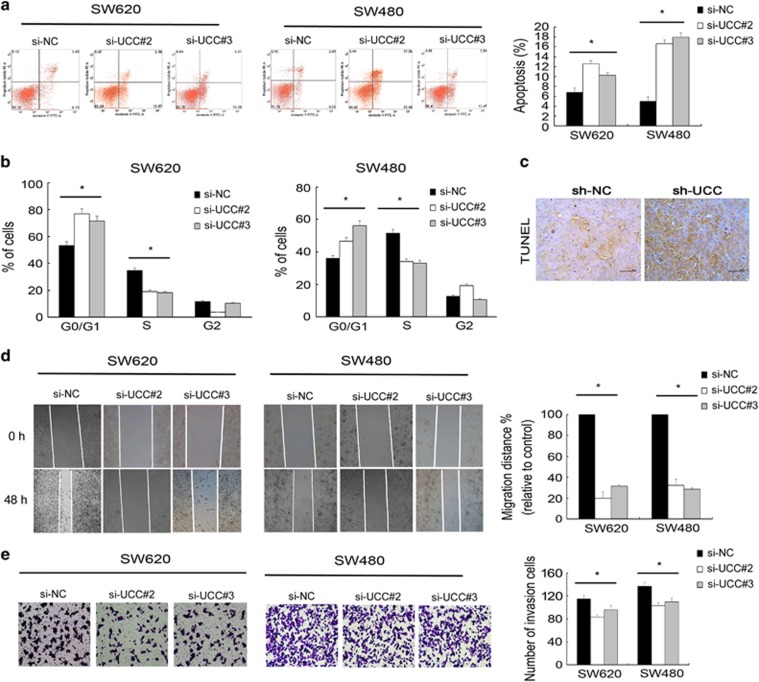
Downregulating *UCC* induces CRC cell apoptosis and G_1_/S arrest and inhibits invasion. (**a**) The effect of *UCC* knockdown on apoptosis of SW620 and SW480 cells was determined by measuring the percentage of Annexin V-stained cells using fluorescence correlation microscopy (FCM). Left: 72 h after treatment with negative control and *UCC* siRNA, Right: ratio of early apoptotic cells was collected and presented in the column chart. (**b**) The cell cycle distribution after knockdown of *UCC* was determined by PI staining and FCM in SW620 and SW480 cells. (**c**) TUNEL assays showed that *UCC* knockdown induced apoptosis of xenograft tumor cells. (**d**) Silencing *UCC* decreased SW620 and SW480 cell mobility. Left: the width of the scratch-wounded cell monolayer was recorded at 0 and 48 h after wounding via photography. Right: the relative migration distance presented in the column chart. (**e**) Downregulation of *UCC* inhibited CRC cell invasion based on Transwell assays. The bars indicate mean±S.D. *P<0.05. All the experiments were repeated three times

To explore the potential mechanisms by which *UCC* enhances CRC cell growth *in vitro*, we analyzed differences in apoptosis and cell cycle distributions among SW620 and SW480 cells between *UCC*-depleted and control conditions via flow cytometry analysis. The percentage of early apoptotic cells was significantly increased in the si-*UCC* groups compared to the control groups (Figure 3a). In addition, significant G_1_/S arrest was observed in *UCC*-silenced cells (Figure 3b). In addition, *UCC* knockdown induced apoptosis of xenograft tumor cells *in vivo*, as determined by TUNEL assays (Figure 3c). These data demonstrated that induction of apoptosis and G_1_/S cell cycle arrest may contribute to *UCC* knockdown-mediated growth inhibition.

To further determine whether *UCC* is associated with the progression of CRC, we analyzed the effect of *UCC* knockdown on invasion of SW620 and SW480 cells. The results of the wound-healing assay showed that knockdown of *UCC* inhibited cell mobility compared with the control treatment (Figure 3d). In addition, Transwell assays indicated that the invasive capacity of the cells was significantly decreased by *UCC* knockdown (Figure 3e).

### Overexpression of *UCC* abrogates CRC proliferation and invasion

We further assessed the biological function of *UCC* by upregulation its expression using pcDNA3.1-*UCC* plasmid vector, focusing on CRC cell line (HCT116) with moderate *UCC* level. *UCC* expression level was significantly elevated after transfection with pcDNA3.1-*UCC* vector (Supplementary Figure S4A). It was implied that overexpression of *UCC* increased the viability of HCT116 cells by MTS assay (Supplementary Figure S4B). Also, colony formation assays and EdU incorporation assays showed that *UCC* upregulation enhanced the proliferative potential of HCT116 cell (Supplementary Figures S4C and D). Taken together, these data indicated that overexpression of *UCC* promoted CRC cell growth.

In addition, we performed flow cytometry to analyze the differences in apoptosis and cell cycle distributions in HCT116 between *UCC*-overexpressed and control group. As expected, the percentage of early apoptotic cells was significantly decreased in the *UCC*-overexpressed groups compared to the control (Supplementary Figure S4E), and the proportion of G_0_/G_1_ was markedly declined after upregulation of *UCC* expression (Supplementary Figure S4F). Collectively, these results suggested that overexpression of *UCC* led to suppression of apoptosis and the proportion of G_0_/G_1_.

Furthermore, the wound healing and Transwell assays were conducted to investigate the biological role of *UCC* in cell invasion. Interestingly, the invasive potential of cells was enhanced in cells transfected with pcDNA3.1-*UCC* plasmid vector (Supplementary Figures S5A and B).

### Inversely correlated expression of *UCC* and miR-143 in CRC

**Figure 4:**
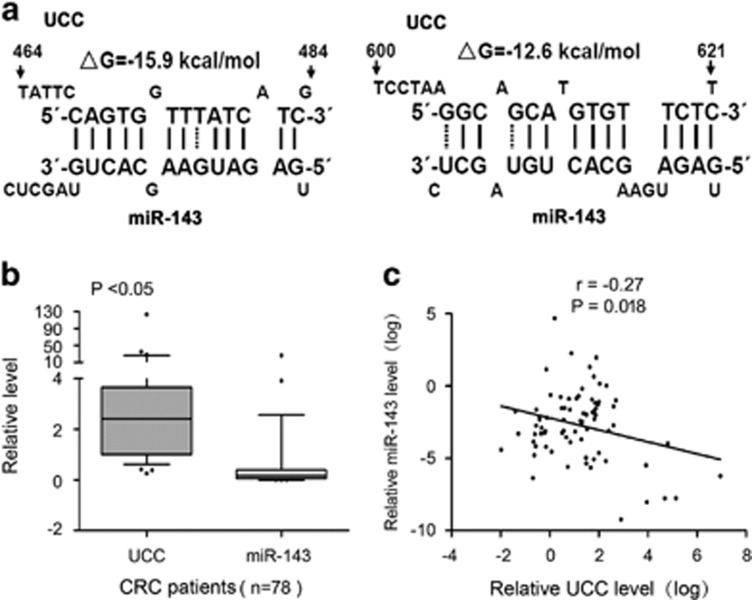
miR-143 is predicted to interact with *UCC*, and miR-143 expression negatively correlates with *UCC* expression. (**a**) Predicted miR-143-binding sites in the *UCC* sequence. The numbers show the distance in nucleotides from the transcriptional start site of *UCC*. (**b**) Relative levels of *UCC* and miR-143 were determined in 78 paired CRC and non-tumor tissues via qRT-PCR. Horizontal lines in the box plots represent the median, the boxes represent the interquartile range and the whiskers represent the 5th and 95th percentiles. The statistical differences between samples were analyzed with paired samples *t*-test (*n*=78, *P*<0.05). (**c**) The expression of *UCC* negatively correlated with that of miR-143 in clinical specimens (*n*=78). *r*=−0.27. *P*=0.018

Accumulating evidence has shown that miRNAs are able to interact with lincRNAs and regulate their expression levels.^17, 18^ Thus, potential miRNA candidates targeting *UCC* were predicted using miRCode and DIANA-LncBase software.^19, 20^ The predicted sites of miR-143 binding to the *UCC* sequence are illustrated in Figure 4a. The level of *UCC* was upregulated in CRC tissues based on qRT-PCR, whereas miR-143 expression was downregulated in the same tumor specimens (Figure 4b). Spearman correlation analysis suggested a negative relationship between *UCC* and miR-143 expression (*r*=−0.27, *P*=0.018; Figure 4c). These results indicate that there might be an inverse correlation between the expression levels of *UCC* and miR-143.

### miR-143 suppresses *UCC* function

**Figure 5:**
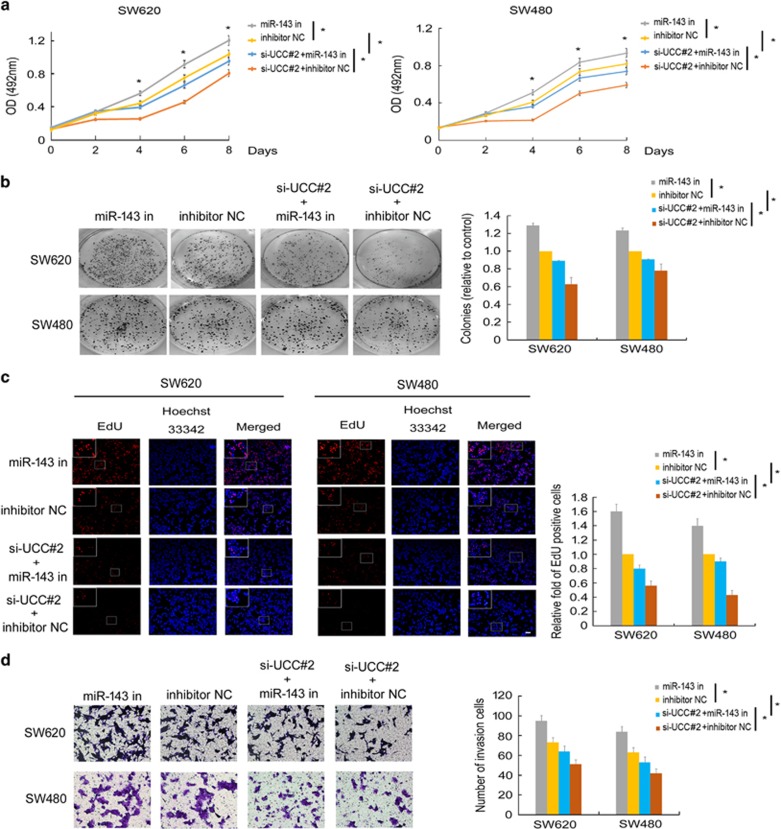
miR-143 inhibits *UCC* function. (**a**) SW620 and SW480 cells were co-transfected with negative control siRNA or si-*UCC#2* and the miR-143 inhibitor, and cell viability was evaluated via MTS assays. (**b**) Colony formation assays of CRC cells after co-transfection with negative control siRNA or si-*UCC#2* and the miR-143 inhibitor. (**c**) EdU incorporation assays of CRC cells after co-transfection with negative control siRNA or si-*UCC#2* and the miR-143 inhibitor. Scale bar: 200 *μ*m. (**d**) Transwell invasion assays of CRC cells after co-transfection with negative control siRNA or si-*UCC#2* and the miR-143 inhibitor. The data represent the mean±S.D. of three independent experiments. *P<0.05 by Student’s *t*-test

We transfected SW620 and SW480 cells with the miR-143 inhibitor or with si-*UCC#2* to study the *UCC*-mediated effects of miR-143 on cell proliferation and invasion. MTS proliferation assays revealed that the miR-143 inhibitor abrogated the effect of si-*UCC#2* in reducing cell viability (Figure 5a). Consistently, the colony formation and EdU incorporation assays confirmed these findings (Figures 5b and c). Transwell invasion assays showed that the miR-143 inhibitor enhanced CRC cell invasion but that si-*UCC#2* inhibited CRC cell invasion. Besides, co-transfection of si-*UCC#2* with the miR-143 inhibitor abolished the repressive effect of si-*UCC#2* on CRC cell invasion (Figure 5d), indicating that miR-143 suppresses *UCC* function.

### *UCC* acts as a competing endogenous RNA by directly binding to miR-143

**Figure 6:**
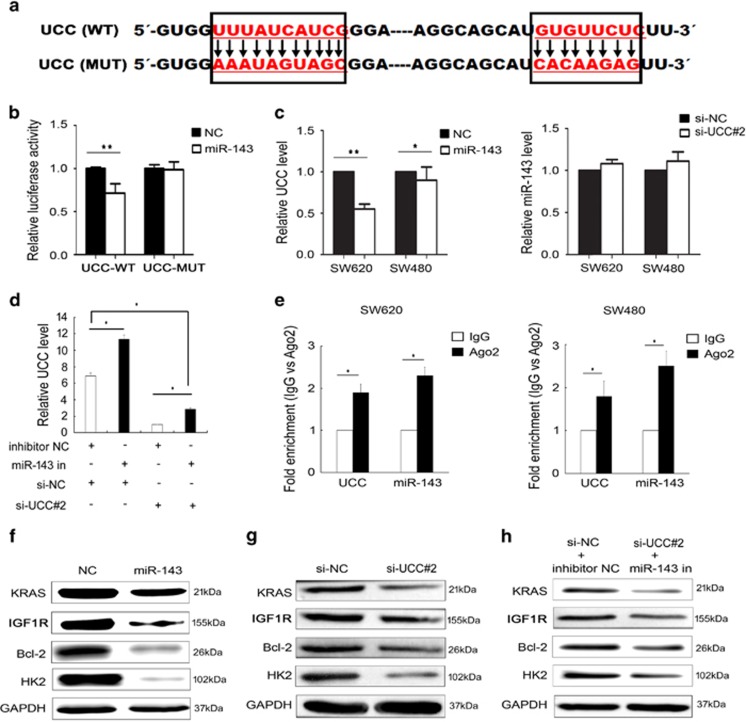
*UCC* is a direct target of miR-143. (**a**) Schematic of the wild-type and mutant psiCHECK-*UCC* constructs. (**b**) Dual-luciferase assays showed a decrease in reporter activity after co-transfection of psiCHECK-*UCC*-WT and miR-143 compared with transfection of miR-143 alone, whereas no significant difference in reporter activity was observed between transfection of miR-143 alone and co-transfection of psiCHECK-*UCC*-MUT and miR-143 in SW620 cells. (**c**) Left: decreased *UCC* expression in cells after transfection of miR-143 mimics. Right: miR-143 expression levels in cells after *UCC* knockdown. (**d**) Relative *UCC* level was investigated in SW620 cells after transfection miR-143 inhibitor and/or si-*UCC*#2. (**e**) Associations of miR-143 and *UCC* with Ago2. SW620 and SW480 cell lysates were collected for RIP using an anti-Ago2 antibody. Detection of miR-143 and *UCC* was performed via qRT-PCR. (**f**) Effect of transfecting SW620 cells with miR-143 mimics on the expression of miR-143 target genes based on western blot. (**g)** Effect of transfecting SW620 cells with si-*UCC* on the expression of miR-143 target genes based on western blot. (**h**) Effect of co-transfecting SW620 cells with si-*UCC* and the miR-143 inhibitor on the expression of miR-143 target genes based on western blot. The bars indicate mean±S.D. (*n*= 3). *P<0.05, **P<0.01 by Student’s *t*-test

To examine the potential lincRNA–miRNA interaction, we subcloned full-length *UCC* or *UCC* harboring a site-directed mutation in the miR-143-binding site into the psiCHECK dual luciferase reporter vector (referred to as *UCC*-WT or *UCC*-MUT, respectively) (Figure 6a). Dual-luciferase assays showed a significant decrease in luciferase activities after co-transfecting cells with miR-143 mimics and the *UCC*-WT expression vector (*P*<0.05, Figure 6b) but not the *UCC*-MUT vector (*P*>0.05, Figure 6b). We further clarified the regulatory relationship between *UCC* and miR-143. Overexpressing miR-143 significantly inhibited *UCC* expression, whereas silencing *UCC* did not affect miR-143 expression (Figure 6c). Inversely, suppressing miR-143 enhanced *UCC* expression. Interestingly, attenuation of *UCC* expression was observed after co-transfection miR-143 inhibitor and si-*UCC#2* when compared to the NC control group (Figure 6d). These results suggest that *UCC* is targeted by miR-143.

Previous studies have demonstrated that miRNAs are present in the form of miRNA ribonucleoprotein complexes that contain Ago2, the key component of the RNA-induced silencing complex (RISC).^21, 22^ Considering that *UCC* is exclusively localized to the nucleus (Figure 1f) and that Ago2 generally interacts with RNAs exported to the cytoplasm, we next performed RNA immunoprecipitation (RIP) using an anti-Ago2 antibody. The Ago2 protein was sufficiently immunoprecipitated from cell extracts. In addition, both *UCC* and miR-143 were enriched by 1.8–2.3-fold following immunoprecipitation using the anti-Ago2 antibody compared to IgG (Figure 6e).

miR-143 has been reported to target and repress KRAS, IGF1R, Bcl-2, and HK2 expression in CRC.^23, 24, 25, 26, 27^ Western blot assays revealed that the expression levels of these genes were dysregulated in CCD841 cells compared to SW620 cells (Supplementary Figure S6) and that forced expression of miR-143 triggered a significant silencing effect on the expression of these genes in SW620 cells, confirming that they are targets of miR-143 (Figure 6f). These repressive effects were also observed in SW620 cells transfected with si-*UCC* (Figure 6g). Furthermore, these effects were maintained by co-transfection with si-*UCC* and the miR-143 inhibitor (Figure 6h). Collectively, these results suggest that *UCC* regulates the target genes of miR-143 by sequestering endogenous miR-143. We provide evidence that *UCC* may act as an endogenous sponge by binding to miR-143, thus abolishing the miRNA-induced repression of its target genes.

## Discussion

Although thousands of lincRNAs were identified recently, functional characterization of lincRNAs has just begun. Functional studies have indicated that some lincRNAs participate human cancer pathogenesis by acting as oncogenes or tumor suppressors.^28, 29^ In the current study, we showed that the novel lincRNA *UCC* is frequently overexpressed in advanced CRC tissues and that *UCC* upregulation correlates with lymph node metastasis and patient outcomes, suggesting a pro-oncogenic activity of *UCC*. This observation is further supported by the results of loss-of-function and gain-of-function approaches. Suppression of *UCC* expression significantly decreased CRC cell growth, induced apoptosis and G1/S arrest, and inhibited invasion, whereas overexpression of this lincRNA had the opposite effects.

miRNAs, which are ~22-nucleotide RNAs with sequence complementarity to the 3′-UTR of mRNAs of target genes, play an important role in gene regulation via translational repression and/or mRNA degradation.^30, 31^ lncRNAs are generally more readily accessible to miRNAs because no proteins are translated from the lncRNA sequence. Several lncRNAs, such as HULC, HOTAIR, HOTTIP, GAS5, and HOST2, have been identified as miRNA targets in various cancers,^17, 18, 32, 33, 34^ and these findings provide further understanding of lncRNA regulation during tumorigenesis.

Using online software, we identified *UCC* as a possible target of miR-143. Generally, miR-143 is downregulated in a variety of tumors, including lung cancer, pancreatic cancer, and melanoma.^13, 14, 15, 16, 35, 36, 37, 38, 39^ As a putative tumor suppressor, miR-143 participates in CRC development and progression by targeting KRAS, IGF1R, Bcl-2, and HK2.^23, 24, 25, 26, 27^ In addition, miR-143 is a plasma miRNA that provides high diagnostic accuracy for early-stage HCC^39^ and is a predictive factor for the response to fluoropyrimidine-based chemotherapy in patients with metastatic CRC,^40^ indicating the clinical relevance of miR-143. Although miR-143 has been experimentally shown to target many protein-coding genes, our data show that miR-143 also targets *UCC*. First, we found a negative correlation between *UCC* and miR-143 expression in clinical CRC specimens. Overexpressing miR-143 reduced *UCC* expression in CRC cells. In addition, we provide evidence that miR-143 targets *UCC* by directly binding to miRNA-binding sites in the *UCC* sequence.

Essentially, a miRNA is bound by a member of the Argonaute family of proteins and confers sequence specificity to a large protein complex. In the cytoplasm, the RNAi machinery uses Watson–Crick base pairing to target the RISC to a specific mRNA and facilitate its degradation.^41, 42^ Alternatively, the Argonaute protein family has been shown to mediate functional RNAi within the nucleus.^43^ A related process is well established in the nucleus of *S*.* pombe*, where instead of targeting cytoplasmic mRNAs for destruction, a small RNA targets the RNA-induced transcriptional silencing complex to the pericentromeric regions of each chromosome and facilitates the generation of heterochromatin.^44, 45^ Ago2 and the RNAi factors Dicer and TRBP were also detected in the human nucleus and can mediate functional RNAi in nucleus.^46^ Moreover, mature miRNAs can be transported from the cytoplasm to the nucleus by importin 8.^47^ That is, there is a primary machinery for Ago2-miRNA-mediated RNA silencing in cell nuclei in humans, which explains why *UCC* primarily localized to the nucleus can physically interact with Ago2. Similar miRNA regulation mechanisms were observed for other nuclear lncRNAs. For instance, MALAT1 is a well-known nuclear lncRNA that can be directly regulated by several miRNAs.^48, 49^

In summary, we have identified that a novel lincRNA, termed *UCC*, is upregulated in human CRC tissues and serves as a negative prognostic factor in CRC patients. Silencing *UCC* inhibits CRC cell proliferation and invasion and induces apoptosis. *UCC* functions as an oncogene in CRC, mechanistically acting by upregulating KRAS and other target genes in part through sponging miR-143.

## Materials and Methods

### Clinical specimens and cell culture

The use of human specimens in this study was sanctioned by the local ethics committee at Sun Yat-sen Memorial Hospital of Sun Yat-sen University (Guangzhou, China). None of the patients received preoperative chemotherapy or radiotherapy. The data collected included age, gender, overall survival, and tumor features such as tumor size, clinical stage, tumor invasion depth, tumor location, and occurrence of distant metastasis. Tumor and adjacent non-tumor tissues were snap-frozen in liquid nitrogen immediately after extraction and stored at −80 °C until total RNA was extracted. The human CRC cell lines SW480, SW620, HCT116, Caco-2, DLD-1, and HT29 and the colonic epithelial cell line CCD841 were obtained from American Type Culture Collection (ATCC, Rockville, MD, USA) and maintained in RPMI-1640 medium (Gibco, Grand Island, NY, USA) supplemented with 10% fetal bovine serum (FBS), 100 U/ml penicillin sodium and 100 mg/ml streptomycin sulfate in a humidified atmosphere (37 °C and 5% CO_2_).

### RNA isolation and qRT-PCR

Total RNA was extracted and purified from tissues and cell lines with Trizol reagent (Life Technologies, Carlsbad, CA, USA) using a standard procedure. After the quality and quantity of the extracted total RNA were confirmed using a NanoDrop ND-1000 spectrophotometer (Thermo Scientific, Rockford, IL, USA), complementary DNA (cDNA) was synthesized using a reverse transcription kit (TaKaRa, Dalian, China) according to the manufacturer’s protocol. In brief, a master mixture containing 1 *μ*l of cDNA sample, 10 *μ*l of SYBR Green qRT-PCR Master Mix (Qiagen, Hilden, Germany) and 1 *μ*l of primers was prepared on ice. The final volume was then adjusted to 20 *μ*l with RNase-free water. All reactions were performed in a Roche LightCycler system (Roche, Basel, Switzerland). Relative expression was calculated using the 2^−ΔΔCt^ method. Each PCR amplification was performed in triplicate to verify the results. The primer sequences used for PCR are listed as follows: *UCC* forward: 5′-GAAAGCATTTTGAAAGCCACTG-3′ and reverse: 5′-GAAACTCACCAACCCAAACCTC-3′ *GAPDH* forward: 5′-GCACCGTCAAGGCTGAGAAC-3′ and reverse: 5′-TGGTGAAGACGCCAGTGGA-3′ *LINC01558* forward: 5′-AGCTGGAGATGTGGTCAACG-3′ and reverse: 5′-ATGGAGCCTTCCCAGTGTTG-3′ *LINC00239* forward: 5′-GTGTGAAGCAAGGGACAGGT-3′ and reverse: 5′-GGGTGCGTCACTTTCCAATG-3′ *HNF1A-AS1* forward: 5′-ACATGACGACCCCACTTCTC-3′ and reverse: 5′-TTGAGTCGTCCATGCCCT TG-3′.

### LncRNA profiling

For lncRNA microarray, RNA purity and integrity was analyzed by Agilent Bioanalyzer 2100 (Agilent, Santa Clara, CA, USA). Qualified total RNA was further purified by RNeasy mini kit (Qiagen) and RNase-free DNase set (Qiagen). Total RNA was then amplified and labeled by Low Input Quick Amp Labeling Kit, One-Color (Agilent), following the manufacturer's instructions. Labeled cRNA were purified by RNeasy mini kit (Qiagen). Each Slide was hybridized with 600 ng Cy3-labeled cRNA using Gene Expression Hybridization Kit (Agilent) in Hybridization Oven (Agilent), according to the manufacturer's instructions. After 17 h hybridization, slides were washed in staining dishes (Thermo Scientific) with Gene Expression Wash Buffer Kit (Agilent), following the manufacturer's instructions. Slides were scanned by Agilent Microarray Scanner (Agilent) with default settings, Dye channel: Green, Scan resolution=3 *μ*m, 20 bit. Data were extracted with Feature Extraction Software 10.7 (Agilent). Raw data were normalized by Quantile algorithm, Gene Spring Software 11.0 (Agilent).

### Subcellular fractionation

The nuclear and cytosolic fractions of SW620 or SW480 cells were separated using the PARIS Kit (Life Technologies) according to the manufacturer’s instructions. RNA was extracted from both fractions. Then, qRT-PCR was performed to assess the expression ratios of specific RNA molecules between the nuclear and cytoplasmic fractions. GAPDH served as the cytosolic control, and U6 served as the nuclear control.

### Cell transfection

The sequence of short-hairpin RNA (shRNA) directed against *UCC* (5′-GGAAGCCCTTGGTAAAGAATTCAAGAGATTCTTTACCAAGGGCTTCC-3′) was ligated into the pLKO.1-Puro vector (TaKaRa). Lentivirus was packaged into HEK 293 cells using Lipofectamine 2000 (Life Technologies) and collected from the supernatant in accordance with the manufacturer’s instructions. Lentiviral particles were used to infect SW620 cells. The synthesized and purified *UCC* gene fragment was inserted into the expression vector pcDNA3.1(+) (Invitrogen, Carlsbad, CA, USA) for overexpression this lincRNA in HCT116 cell line. Stable cell lines were established via puromycin selection and then used for subsequent *in vitro* and *in vivo* experiments. For transient transfection assays, miR-143 mimics, a miR-143 inhibitor, small interfering RNA (siRNA) duplexes (si-*UCC*#1, si-*UCC*#2 and si-*UCC*#3), and negative control (NC) RNA duplexes for miRNA mimics, the miR-143 inhibitor or the siRNAs were synthesized (Ribobio, Guangzhou, China). The siRNA sequence for si-UCC were si-*UCC#1*, 5′-GGAGAGACUGCUCUCUCAU-3′, si-*UCC#2*, 5′-GGAAGCCCUUGGUAAAGAA-3′ and si-*UCC#3*, 5′-GCUUGAUGUUAGAGCUUAA-3′. These oligonucleotides were transfected into SW620 and SW480 cells using Lipofectamine RNAiMAX (Invitrogen) according to the manufacturer’s protocol.

### Cell growth assay

For cell proliferation assay, the MTS assay from Promega (Madison, WI, USA) (CellTiter 96 AQueous One Solution Cell Proliferation Assay) was used following manufacturer’s instruction. Briefly, cells in a 96-well plate were incubated in a humidified 5% CO_2_ chamber after transfection with indicated siRNAs or vector, followed by addition of 20 *μ*l CellTiter 96 AQueous One Solution and 1–4 h incubation in humidified 5% CO_2_ chamber. The absorbance at 492 nm was recorded. The assay was performed using six replicates. For colony formation assay, the cells transfected with indicated oligonucleotides for 24 h or the stable HCT116 cells, and were seeded in six-well plates. After 14 day incubation, the number of clones were counted and analyzed.

### Wound healing assay

Cells were incubated with normal cell growth medium in six-well plates. Once cultures reached 85% confluency, the cell layer was scratched with a 10 *μ*l sterile pipette tip and washed with culture medium, then exchanged with medium containing 1% FBS cultured for 48 h. To prevent cell proliferation, which could confound the analysis of cell migration into the wound, cells were preincubated with mitomycin C (10 *μ*g/ml) for 1 h at 37 °C. At different time points (0, 48 h), images of the plates were acquired using a microscope.

### Transwell assays

Cell invasion assays were carried out using 24-well Transwell chambers with 8 *μ*m pore size polycarbonate membrane (Costar, Corning, NY, USA). Briefly, the lower chamber was filled with 600 *μ*l RPMI 1640 containing 20% FBS. Cells were trypsinized, counted and re-suspended in serum-free RPMI 1640. Cells (2 × 10^4^) in 200 *μ*l serum-free RPMI were added to the upper chamber. The cells were allowed to invade for 24 h at 37 °C before fixing. The non-invaded cells were removed from the upper surface of the membrane by scraping with a cotton swab. Cells on the bottom surface of the membrane were fixed with 95% ethanol and then stained with 1% crystal violet in methanol/PBS. Invasion was assessed by counting the number of stained cell nuclei from five randomly fields per filter in each group at × 200 magnification using a Zeiss (Melville, NY, USA) microscope system.

### Cell cycle distribution and apoptosis analysis

To detect the effect of downregulation of UCC on cell cycle distribution and apoptosis, flow cytometry assay was performed. For cell cycle distribution analysis, SW620 and SW480 cells collected at 72 h after transfection with si-UCC, together with stable transfected HCT116 cells, were trypsinized and fixed with ice-cold 70% ethanol for 18 h at 4 °C. The fixed cells were stained with 50% mg/ml Propidium iodide (PI) (BD Pharmingen, San Diego, CA, USA) and 50 mg/ml RNase and then analyzed using a flow cytometer (BD Pharmingen). For apoptosis analysis, si-UCC transfected SW620 and SW480 cells harvested at 72 h after transfection, as well as stable transfected HCT116 cells, were stained with FITC-Annexin V and PI and then analyzed using a flow cytometer. Triplicate experiments with triplicate samples were performed.

### Sodium dodecyl sulfate-polyacrylamide gel electrophoresis and western blot assays

Total cell lysate was prepared with a buffer containing 20 mM Tris/HCl, pH 7.4, 300 mM NaCl and 1% Triton X-100. After centrifugation (10 000 × *g*, 10 min, 4 °C), the supernatant was separated via 10% sodium dodecyl sulfate-polyacrylamide gel electrophoresis, transferred to a PVDF membrane, and subjected to western blot analysis utilizing various antibodies. Antibodies recognizing KRAS (ab180772, dilution: 1/200), Bcl-2 (ab59348, dilution: 1/500), HK2 (ab37593, dilution: 1/200), IGF1R (ab39675, dilution: 1/500), and GAPDH (ab9485, dilution: 1/2500) were purchased from Abcam (Cambridge, MA, USA).

### Immunocytochemistry and immunohistochemistry assays

At 48 h after transfection, the 5-ethynyl-2′-deoxyuridine (EdU) incorporation assay was performed using the Cell-Light EdU Apollo567 *In Vitro* Imaging Kit (Ribobio) according to the manufacturer’s instructions. The xenograft tumor tissues were harvested in 4% formaldehyde buffered with phosphate-buffered saline, embedded in paraffin and then sectioned. An antibody against Ki-67 (#9449, Cell Signaling Technology, Danvers, MA, USA, dilution: 1/400) was used for immunohistochemical analyses. Immunoreactivity in the sections was detected using a horseradish peroxidase (3,3′-diaminobenzidine substrate) kit (BioGenex, Fremont, CA, USA). The slides were then counterstained with hematoxylin, dehydrated and mounted. Terminal deoxynucleotidyl transferase-mediated deoxyuridine triphosphate nick-end labeling (TUNEL) (Roche) assays were carried out according to the manufacturer’s protocol.

### The subcutaneous xenotransplantation model

Animal experiments were approved by the Sun Yat-sen University Institutional Animal Care and Use Committee and were conducted following the animal treatment policies of Sun Yat-sen University in accordance with the National Institutes of Health guidelines. After 1 × 10^6^ cells were subcutaneously injected into the back flank of 5-week-old female BALB/C nude mice (*n*=5 per group), tumor growth was examined every 5 days for 4 weeks. The tumor volume was calculated according to the following equation: volume=length × width^2^ × 0.5.

### Dual-luciferase reporter assay

The human *UCC* 3′-UTR luciferase reporter construct (*UCC*-WT) was generated by cloning *UCC* mRNA 3′-UTR sequence into downstream of psiCHECK luciferase reporter vector (Promega). The miR-143 target site-mutation *UCC* 3′-UTR luciferase reporter (*UCC*-MUT) construct was generated by employing direct-site mutagenesis using mutation primers that mutate the miR-143-binding site. Nucleotide sequence of the constructs were confirmed by DNA sequencing. SW620 cells were seeded at 3 × 10^4^ cells per well into 24-well plates and allowed to settle overnight. Next day, cells were co-transfected with wild-type or mutant reporter plasmids and miR-143 mimics. Twenty-four hours after co-transfection, the relative luciferase activity was measured using the dual-luciferase reporter assay system (Promega). Data were normalized by dividing Firefly luciferase activity with that of Renilla luciferase. For each luciferase construct, three independent transfections were performed (each in triplicate). Fold increase was calculated by defining the activity of the psiCHECK-Control vector as 1.

### RNA immunoprecipitation

RIP was performed using the EZ-Magna RIP Kit (Millipore, Billerica, MA, USA) according to the manufacturer’s instructions. Briefly, cells were collected and lysed in complete RIP lysis buffer. Then, the cell extract was incubated with RIP buffer containing magnetic beads conjugated to a human anti-Ago2 antibody (Millipore). Samples were incubated with proteinase K with shaking to digest proteins; subsequently, immunoprecipitated RNA was isolated. The RNA concentration was measured using a NanoDrop spectrophotometer, and RNA quality assessed using a bioanalyzer (Agilent). Afterwards, purified RNA was subjected to qRT-PCR analysis.

### Statistical methods

Statistical analysis was performed using SPSS 13.0 statistical software (SPSS, Inc. Chicago, IL, USA). All numerical data are presented as the means±S.D. for multiple samples, except for the relative *UCC* level in patients with/without lymph node metastasis or advanced Dukes’ stage, which is presented as median with range, because there are several samples with very high expression levels. The paired sample *t*-test was used to evaluate the differences in lncRNA expression between the paired groups. The chi-square test (*χ*^2^ test) or Mann–Whitney test was used for non-parametric variables, and (two-tailed) Student’s *t*-test or one-way analysis of variance (ANOVA) was used for parametric variables. Survival was calculated using the Kaplan–Meier method and was analyzed via the log-rank test. A *P-*value of 0.05 or less was considered significant.

## Figures and Tables

**Table 1:** Characteristics of 78 pancreatic ductal adenocarcinoma patients

**Characteristics**	**Patients frequency (%)**	**UCC**		***P*****-value**
		**Low**	**High**	
Gender	78	28	50	
Male	39 (50%)	13	26	0.637
Female	39 (50%)	15	24	
*Age (year)*				
		56.82±2.43	60.62±2.17	0.271
*Lymph node metastasis*				
Absent	46 (59%)	25	21	<0.001^**^
Present	32 (41%)	3	29	
*Distant metastasis*				
Absent	71 (91%)	26	45	0.672
Present	7 (9%)	2	5	
*Dukes’ stage*				
A/B	43 (55%)	24	19	<0.001^**^
C/D	35 (45%)	4	31	

Chi-square test. ^**^P<0.001.
