# Supplementary material for: The novel long intergenic noncoding RNA UCC promotes colorectal cancer progression by sponging miR-143
Source: Cell Death Dis. 2017 May 11;8(5):e2778–. doi: 10.1038/cddis.2017.191 (PMC5520712; doi:10.1038/cddis.2017.191)
Supplement: Supplementary Information [file cddis2017191x1.docx]

**Supplementary Figure Legend**

**Supplementary Figure S1.** lincRNAs are systemically dysregulated in CRC tissues. Heat map representing unsupervised hierarchical clustering of mRNAs (**A**) and lincRNAs expression level (**B**) in CRC tissues (T) compared with adjacent non-tumor tissues (N). (**C**) High resolution of the heat map showing *UCC* expression in the microarray analysis. (**D**) Schematic of genomic location of *UCC*.

**Supplementary Figure S2.** GO and KEGG analysis indicated most genes of microarray were involved in cell proliferation as well as cell death control. (**A**) Molecular function. (**B**) Cellular component. (**C**) Biological process. (**D**) Signaling pathway.

**Supplementary Figure S3.** The expression levels of the candidate lincRNAs determined by qRT-PCR in additional 8 pairs of CRC and non-tumor tissues. The relative expression levels of *UCC* (**A**), *LINC01558* (**B**), *HNF1A-AS1* (**C**) and *LINC00239* (**D**) were detected.

**Supplementary Figure S4.** Overexpression of *UCC* abrogates CRC cell growth *in vitro*. (**A**) *UCC* expression levels were effectively up-regulated by pcDNA3.1-*UCC* plasmid vector in HCT116 cells. (**B**) Growth curves of stable transfected HCT116 cells after up-regulation *UCC* were determined via MTS assays. (**C**) Colony formation assay indicated that the anchorage-independent growth of HCT116 cells was enhanced after overexpression of *UCC*. (**D**) *Upper*: Cell proliferation was evaluated using EdU incorporation assays. Proliferating cells were labeled with EdU. *Lower*: Relative ratio of EdU positive cells presented in the column chart. (**E**) The effect of *UCC* up-regulation on apoptosis of HCT116 cells was evaluated using fluorescence correlation microscopy. *Upper*: 72 hours after treatment with negative control and *UCC*-overexpressed vector, *Lower*: Ratio of early apoptotic cells was collected and presented in the column chart. (**F**) The cell-cycle distribution after overexpression of *UCC* was assessed by flowcytometric analysis. The data represent the mean ± S.D. of three independent experiments. ^*^p<0.05 by Student’s t-test.

**Supplementary Figure 5.** Up-regulating *UCC* enhances invasion. (**A**) Enhancement of *UCC* expression promoted HCT116 cell mobility. *Left*: The width of the scratch-wounded cell monolayer was recorded at 0 and 48 hours after wounding via photography. *Right*: The relative migration distance presented in the column chart. (**B**) Overexpression of *UCC* contributed to CRC cell invasion based on Transwell assays. The bars indicate mean ± S.D. ^*^p<0.05. All the experiments were repeated in triplicate.

**Supplementary Figure S6.** The levels of miR-143 targets were detected by Western blot in SW620 cells and colonic epithelial cell line CCD841.
