# Supplementary figures and images for: The novel long intergenic noncoding RNA UCC promotes colorectal cancer progression by sponging miR-143
Source: Cell Death Dis. 2017 May 11;8(5):e2778–. doi: 10.1038/cddis.2017.191 (PMC5520712; doi:10.1038/cddis.2017.191)

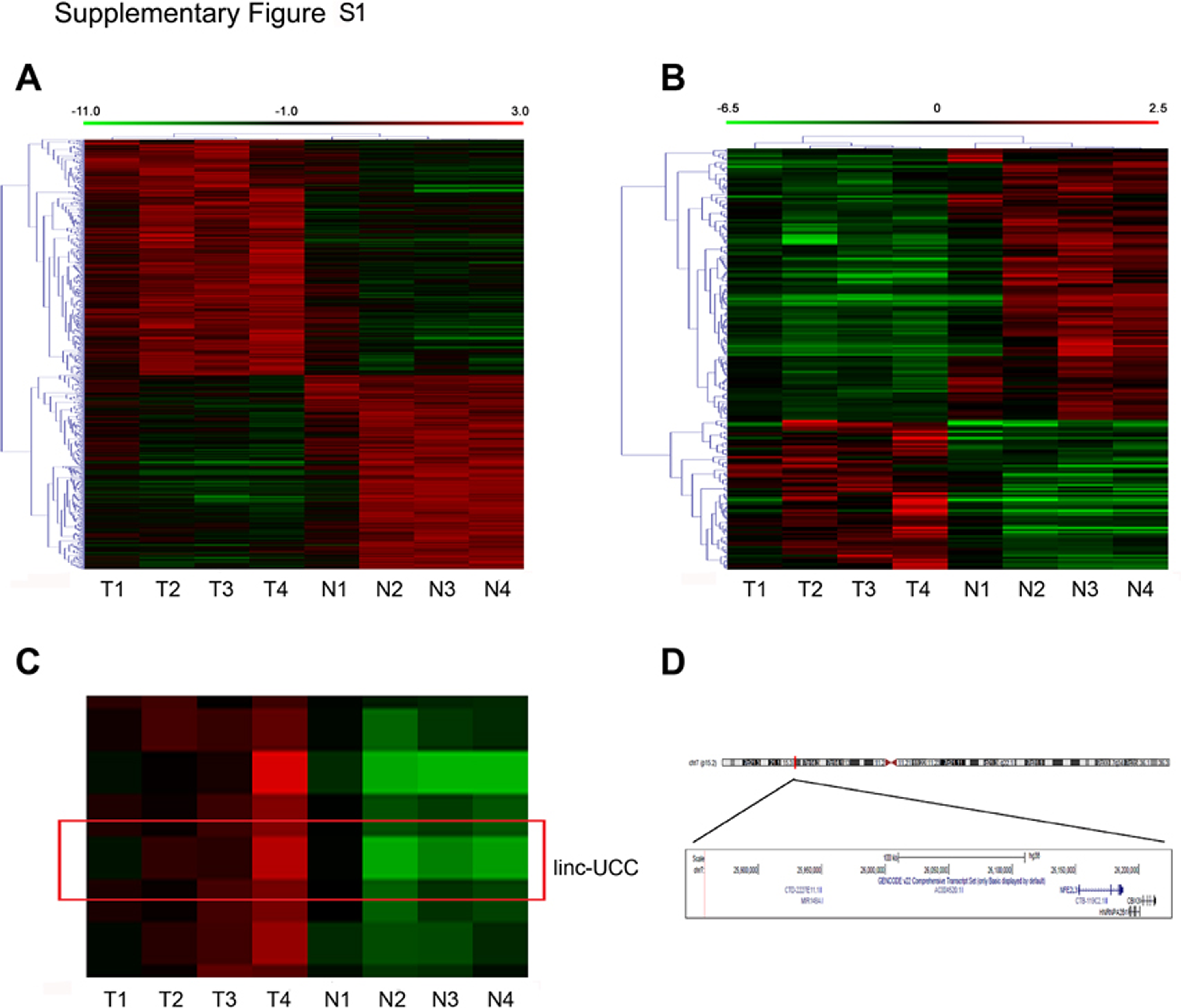

Supplement: Supplementary Figure 1 [file cddis2017191x2.tif]

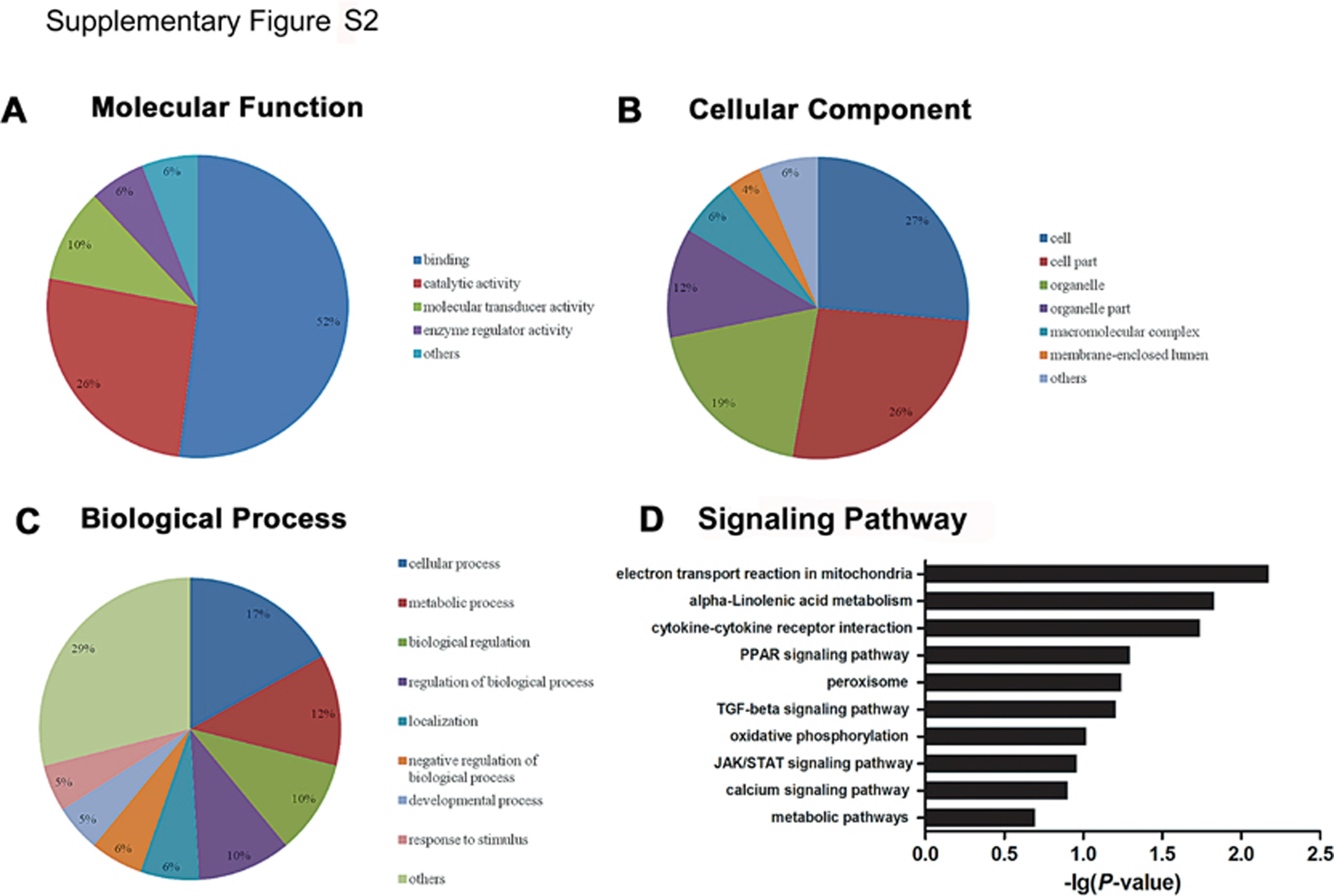

Supplement: Supplementary Figure 2 [file cddis2017191x3.tif]

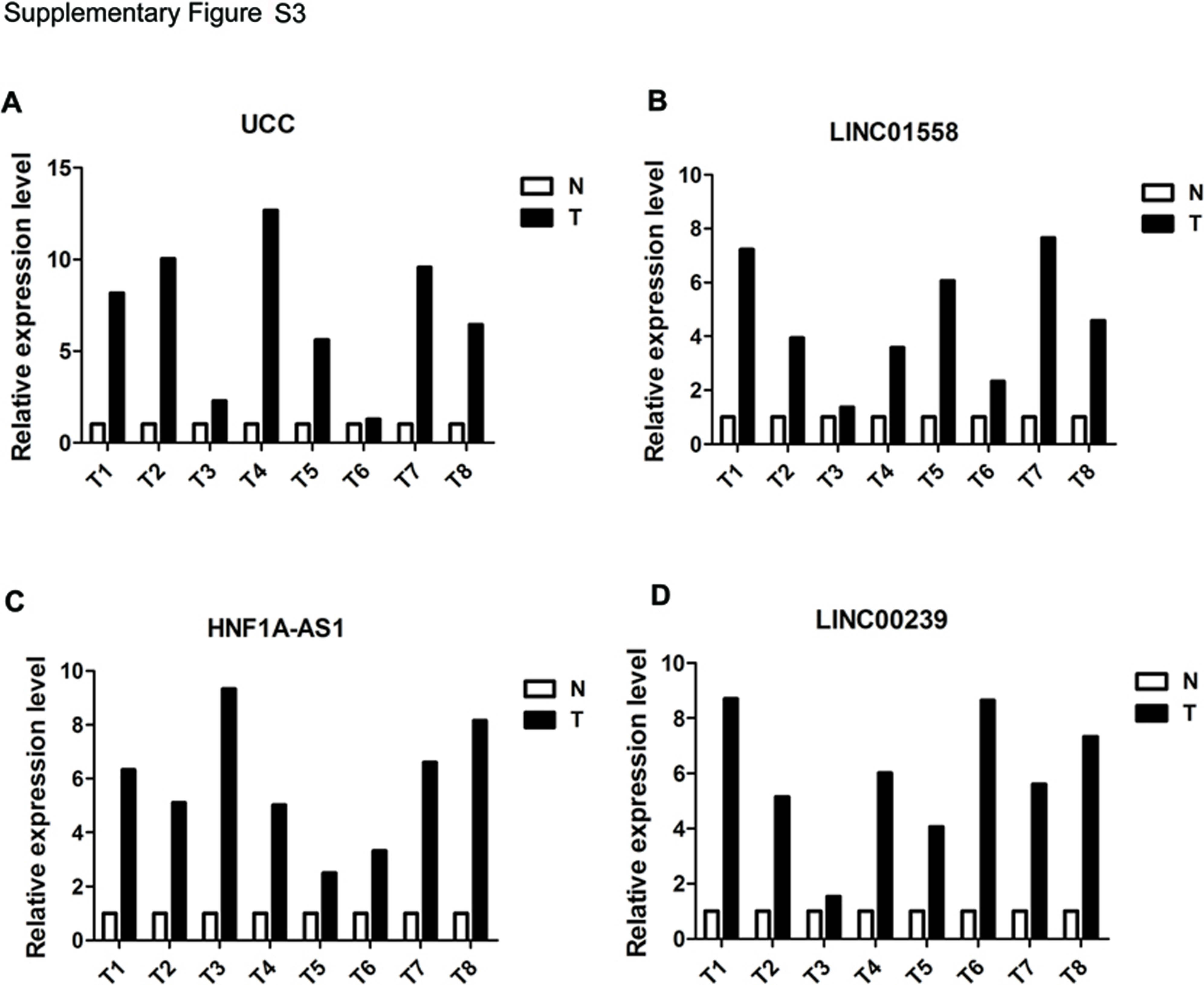

Supplement: Supplementary Figure 3 [file cddis2017191x4.tif]

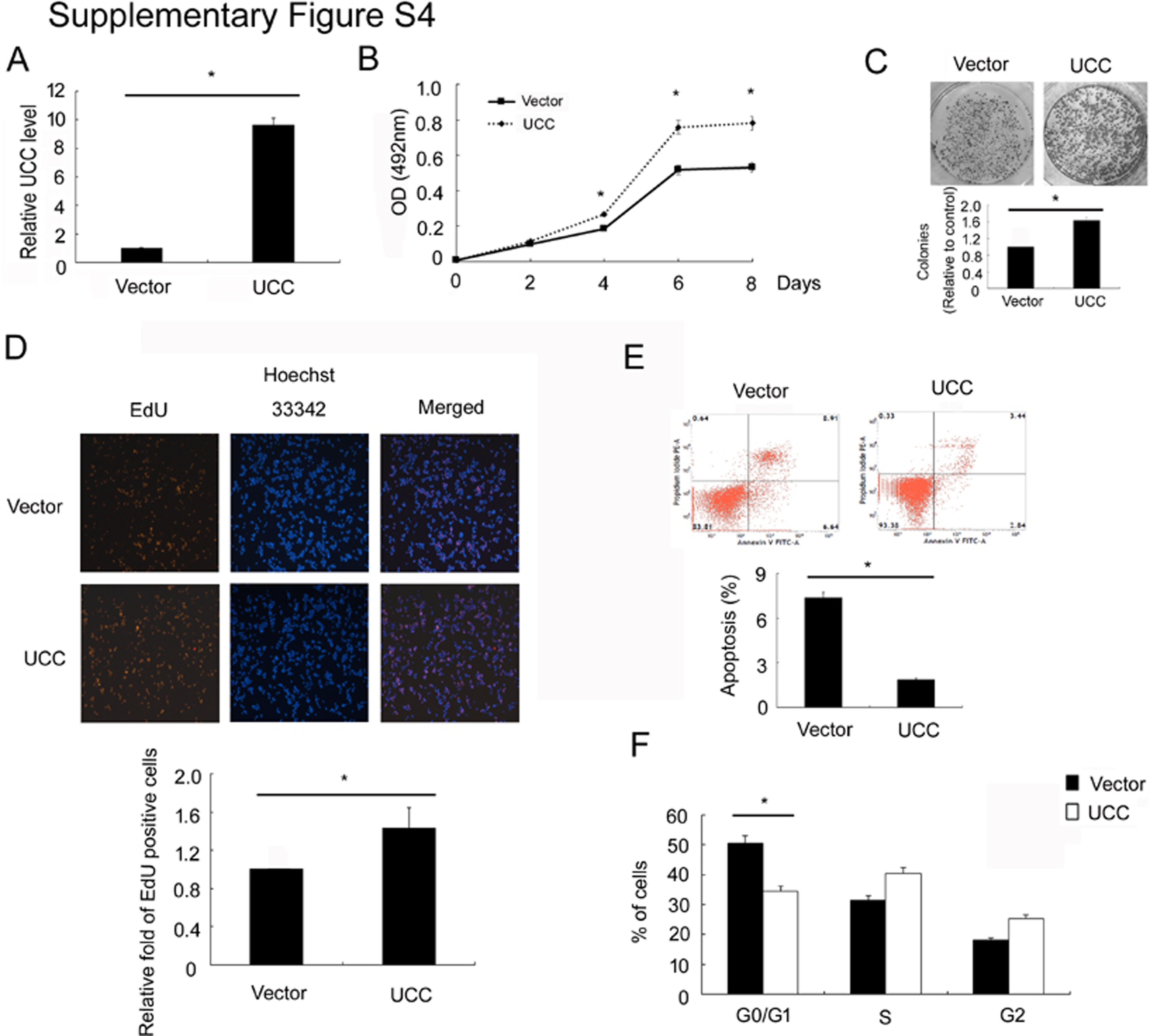

Supplement: Supplementary Figure 4 [file cddis2017191x5.tif]

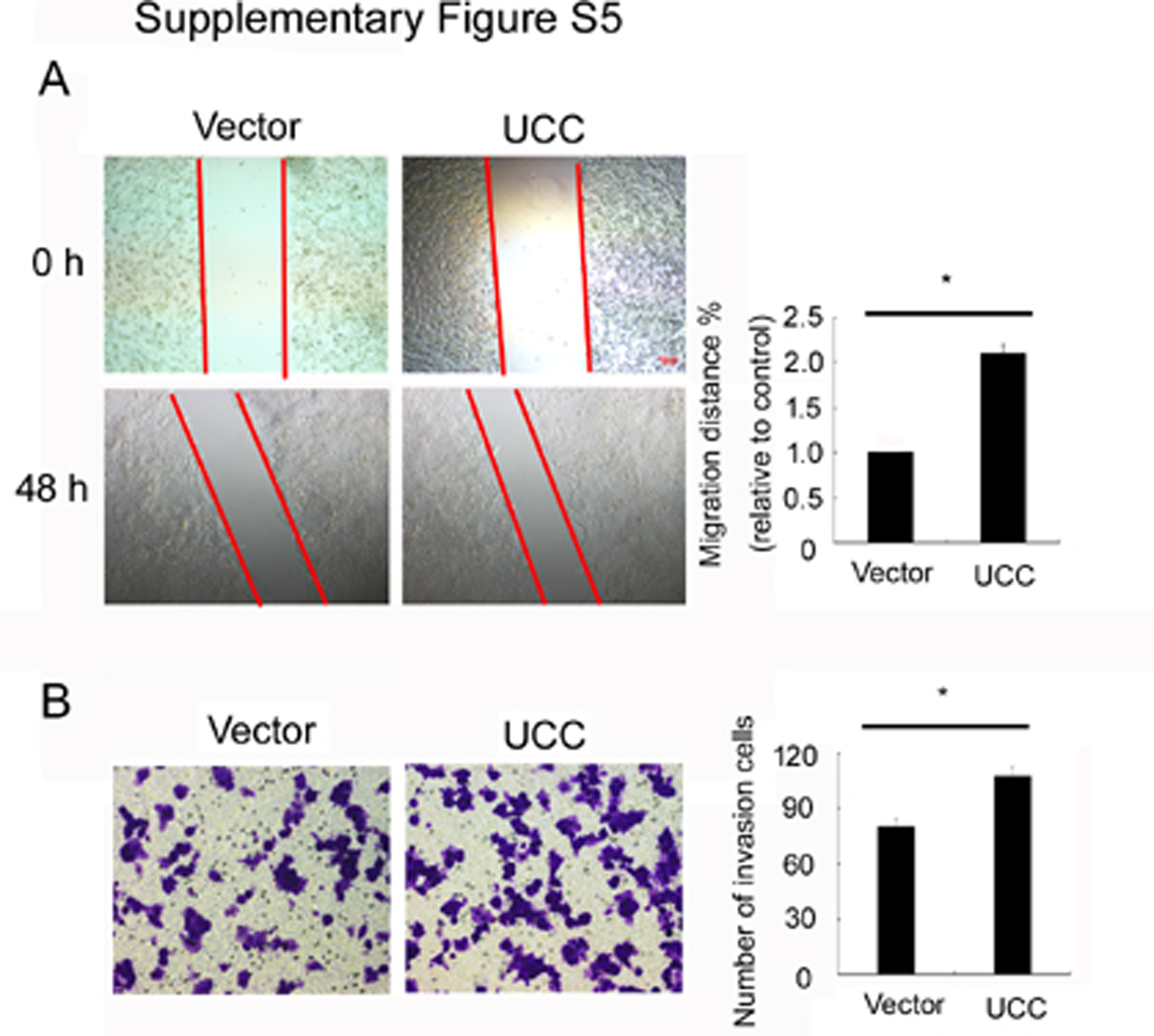

Supplement: Supplementary Figure 5 [file cddis2017191x6.tif]

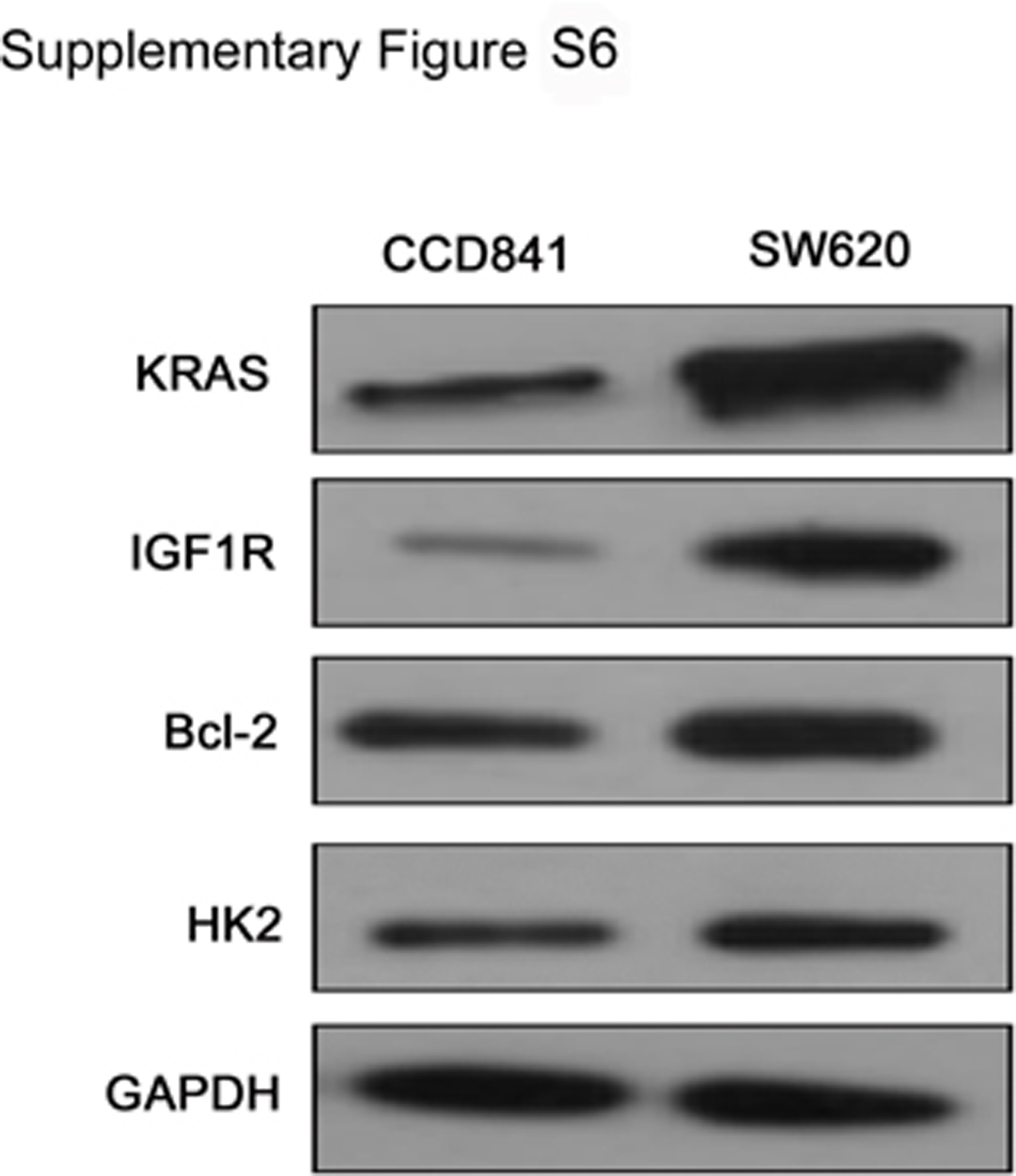

Supplement: Supplementary Figure 6 [file cddis2017191x7.tif]
